# Mutation-Derived Neoantigens for Cancer Immunotherapy

**DOI:** 10.3389/fimmu.2019.01856

**Published:** 2019-08-07

**Authors:** John C. Castle, Mohamed Uduman, Simarjot Pabla, Robert B. Stein, Jennifer S. Buell

**Affiliations:** Agenus Inc., Lexington, MA, United States

**Keywords:** cancer, mutations, neoantigens, immunotherapy, therapeutic vaccine, TCR

## Abstract

Mutation-derived neoantigens distinguish tumor from normal cells. T cells can sense the HLA-presented mutations, recognize tumor cells as non-self and destroy them. Therapeutically, immunotherapy antibodies can increase the virulence of the immune system by increasing T-cell cytotoxicity targeted toward neoantigens. Neoantigen vaccines act through antigen-presenting cells, such as dendritic cells, to activate patient-endogenous T cells that recognize vaccine-encoded mutations. Infusion of mutation-targeting T cells by adoptive cell therapy (ACT) directly increases the number and frequency of cytotoxic T cells recognizing and killing tumor cells. At the same time, publicly-funded consortia have profiled tumor genomes across many indications, identifying mutations in each tumor. For example, we find basal and HER2 positive tumors contain more mutated proteins and more TP53 mutations than luminal A/B breast tumors. HPV negative tumors have more mutated proteins than HPV positive head and neck tumors and in agreement with the hypothesis that HPV activity interferes with p53 activity, only 14% of the HPV positive mutations have TP53 mutations vs. 86% of the HPV negative tumors. Lung adenocarcinomas in smokers have over four times more mutated proteins relative to those in never smokers (median 248 vs. 61, respectively). With an eye toward immunotherapy applications, we review the spectrum of mutations in multiple indications, show variations in indication sub-types, and examine intra- and inter-indication prevalence of re-occurring mutation neoantigens that could be used for warehouse vaccines and ACT.

## Introduction

Cells of the immune system recognize and lyse tumor cells. Mutation neoantigens are critical for tumor control: T cells recognize mutant peptides bound to MHC alleles on tumor cells both in mice and humans (1) and tumor mutational burden (TMB) predicts tumor response to anti-CTLA4 (2) and anti-PD1 treatment (3). Tumors that become resistant to pembrolizumab, an anti-PD1-therapy immunotherapy, often contain mutations in immune-related genes, including in interferon-receptor–associated Janus kinases and the antigen-presenting protein beta-2-microglobulin, suggesting that anti-PD1 therapeutic activity is mediated through neoantigen presentation and recognition (4). Individualized vaccination using autologous tumor lysate (5) or autologous tumor-derived heat shock protein-peptide complexes (6) imparted tumor-specific T-cell responses and vaccination with synthetic mutation-encoding peptides or nucleic acids imparted mutation-specific responses in humans and mice (7–9). T cells have been discovered that recognize mutant KRAS neoantigens; transfer of these T cells led to tumor regression in multiple lesions followed by escape in a separate lesion after genetic deletion of the peptide presenting HLA locus (10). Together, these demonstrate that neoantigens encoding mutations can mediate the tumor-focused immune response and can be exploited as an exquisitely tumor-specific therapeutic target.

The Cancer Genome Atlas (TCGA) is a comprehensive effort to understand the molecular basis of cancer (11). TCGA member organizations have profiled hundreds of individual tumors in each of many indications, including the identification of somatic mutations present in each tumor. The mutation profiles are available for public download for further analysis. In addition to analysis of individual tumors, intra- and inter-indication analyses pinpoint re-occurring mutations (12). Mutation frequencies can be divided into sub-populations and the immunogenicity of each mutation can be predicted (13–16). Here, we examine mutations in cancer populations, compare subgroups such as smokers and non-smokers, identify re-occurring mutations, and predict HLA binding of mutation-containing peptides.

## Materials and Methods

TCGA datasets: protein mutations, gene expression, and medical annotation including lung cancer smoking status, head and neck cancer HPV status, colorectal microsatellite status and breast cancer PAM-50 assignment were downloaded from the UCSC Cancer Genomics Browser (17) on April 24, 2015. TCGA tumor mutations were downloaded from the GDC Data Portal (https://portal.gdc.cancer.gov/) on May 3, 2017. Missense mutations were mapped to human reference genome GRCh37 and filtered for those mutations present in at least two tumor samples.

The number of samples for each type: AML (197), thyroid (427), renal chromophobe (66), ovarian (142), breast (982), prostate (425), lower grade glioma (527), glioblastoma (291), renal papillary cell (113), uterine carcinosarcoma (57), renal clear cell (213), colon (224), rectum (81), endometrial (194), stomach (91), head and neck (509), cervical (40), bladder (238), pancreatic (144), lung adenocarcinoma (543), lung squamous cell (178), and melanoma (369).

HLA affinity calculation: for each mutation, we calculated the binding of all possible 8, 9, and 10 amino acid mutation-containing peptides to 23 common HLA alleles using NetMHCpan version 3.0 (14).

## Results

**Figure 1:**
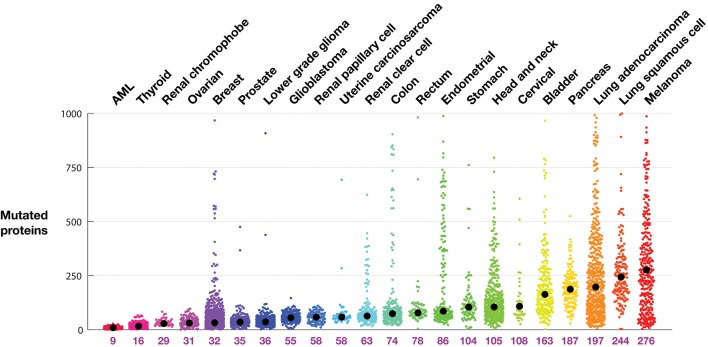
Tumor mutational burden (TMB) in each tumor from TCGA profiles. Tumors are grouped according to indication. Each colored dot represents one tumor. The indication median is indicated by a black circle and listed below the plot. TMB is defined as the number of proteins with a non-synonymous mutation.

Tumor mutational burden by indication: rather than examine mutation rates (18), Figure 1 shows the TMB as the number of proteins with non-synonymous point mutations in a tumor, grouped by cancer indication, along with the indication-specific median. As expected, AML and thyroid tumors have few mutated proteins, with medians of 9 and 16, respectively. Melanoma and lung cancers have the most, with medians of 276, 244, and 197, respectively.

The intra-indication TMB burden varies considerably. The pancreatic tumors profiled in this dataset, for example, contain at least 30 mutated proteins. Conversely, there are melanoma, lung adenocarcinoma, stomach, and head and neck tumors with very few mutations. From the organs known to develop microsatellite instability (MSI) related tumors, including colon, stomach, and endometrial (19), are many tumors with extremely high numbers of mutations. Other indications with high median mutational burden show long tails (populations of tumors with many mutations), in particular melanoma and lung adenocarcinoma, but also lung squamous, bladder, and head and neck tumors. While MSI tumors are uncommon in breast cancer (20), there is curiously a clear population of breast tumors with significantly more mutations.

**Figure 2:**
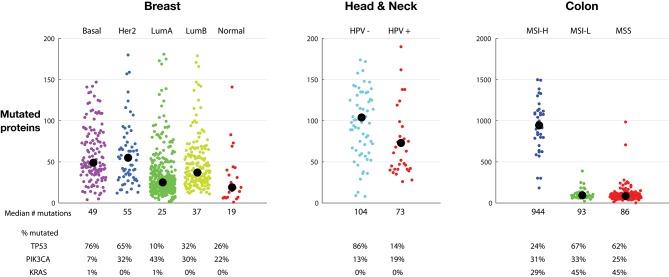
Tumor mutations burden (TMB) in sub-classes of breast tumors **(Left)**, head and neck tumors **(Middle)**, and colon tumors **(Right)**. Each colored dot represents one tumor. Medians are listed and indicated by a black circle. The percentage of tumors with TP53, PIK3CA, and KRAS mutations is listed. The vertical scale of the colon tumor plot is different from the scale in the breast and head and neck tumor plots. TMB is defined as the number of proteins with a non-synonymous mutation.

Figure 2 shows tumor mutations from three indications, each divided into subclasses. Breast tumors can be subdivided into five categories using TCGA gene expression to assign each tumor to the PAM-50 classes: basal, HER2 positive, luminal A, luminal B, and normal (21). The median and spread of mutations across each class varies considerably. Basal tumors, a class highly overlapping with the triple negative classification, and HER2 positive tumors have the highest median number of mutated proteins, 49 and 55, respectively, and a similar broad distribution extending to almost 200 mutations. Normal breast tumors have the lowest median, 19 mutations, while luminal A tumors show a tight symmetrical distribution around the median, 25 mutations. Interestingly, the percentage of tumors containing p53 mutations roughly tracks the median number of mutations in each class, highest in basal and HER2 positive tumors and lowest in luminal A tumors. The percentage of tumors with PIK3CA tumors is by far lowest in the basal group and highest in the luminal A group.

Head and neck tumors comprise a large tumor class, including tumors in the oropharyngeal, oral cavity, oropharynx, hypopharynx, and larynx. Some head and neck tumors, particularly oropharyngeal tumors located in the tonsils and base of the tongue, are caused by HPV infection (22). HPV16 virus can integrate into the genome, and the resulting HPV16 E6 and E7 proteins interfere with activity of endogenous p53 and RB1 protein activity, respectively. Figure 2, middle, clearly shows a marked difference in mutation number: HPV negative tumors have, on average, more mutations than HPV positive tumors: 104 vs. 73 median proteins with non-synonymous point mutations. In agreement with the hypothesis that HPV activity interferes with p53 activity, only 14% of the HPV positive mutations have p53 mutations, vs. 86% of the HPV negative tumors. This suggests that the presence of HPV removes the need to mutate p53.

Figure 2, right, shows colon tumors sub-classified into microsatellite instability high (MSI-H), microsatellite instability low (MSI-L), and microsatellite stable (MSS) classes. Endometrial and stomach tumor sub-classes have similar distributions (not shown). As expected, the MSI-H tumors have a much higher mutation number than MSI-L and MSS tumors. In colon tumors, the MSI-H tumors contain a median of 944 mutations vs. 93 and 86 in the MSI-L and MSS tumors, respectively. The number of tumors with PIK3CA mutations is similar across the three sub-groups. However, similar to the HPV positive tumors, the percentage of MSI-H tumors with p53 mutations is much lower, here suggesting that the MSI-H status lessens the need of p53 mutations for oncogenesis. Likewise, the frequency of KRAS mutations is lower in the MSI-H group, suggesting that the MSI-H phenotype also lessens the need for the KRAS driver activity.

**Figure 3:**
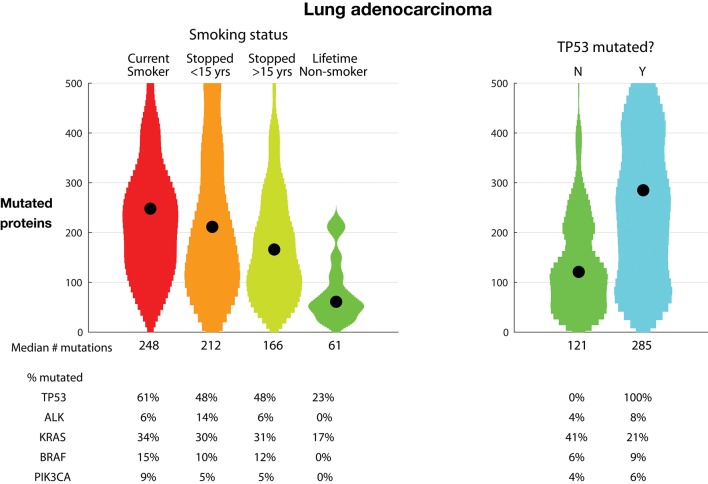
Tumor mutational burden (TMB) in lung adenocarcinoma. Plots show distributions as violin plots with medians indicated as a point. **(Left)** TMB vs. smoking status. **(Right)** TMB vs. TP53 mutational status. TMB is defined as the number of proteins with a non-synonymous mutation.

Figure 3, left, examines the relationship between TMB and smoking in lung adenocarcinoma as reported in the TCGA dataset. There is a dramatic and clear correlation between smoking and TMB. Tumors from never-smokers have a median of 61 mutated proteins; tumors from patients who stopped smoking over 15 years ago have a median of 166, tumors from patients who stopped smoking <15 years ago have a median of 212, and tumors from current smokers have a median of 248 mutated proteins. Recent clinical trials have shown increased benefit of anti-PD1 and anti-CTLA4 antibodies for the treatment of non-small-cell lung cancer (NSCLC) tumors with high TMB, defined as tumors with >10 mutations per megabase or as tumors with great than a median of 158 mutations (23, 24). In the TCGA dataset, these cutoffs eliminate most never-smokers, enriching for current and recent smokers.

The percentage of tumors with TP53 protein mutations is almost three-fold higher in current smokers than in never-smokers, 61% vs. 23%, respectively. Further examining the association of TP53 mutations, Figure 3, right, shows the relationship between TP53 mutations and TMB. The tumors with TP53 mutations have over twice the number of mutated proteins compared to tumors with non-mutated TP53, 285 vs. 121 mutated proteins, respectively. Conversely, the rate of mutated KRAS is almost twice as large in the TP53 non-mutated tumors, 41% vs. 21%, respectively. This occurs despite the lowest rate of mutated KRAS in never-smoker tumors, which have lower TMB and TP53 mutation rates. This suggests that oncogenic dependency on TP53 and KRAS mutations is orthogonal.

**Figure 4:**
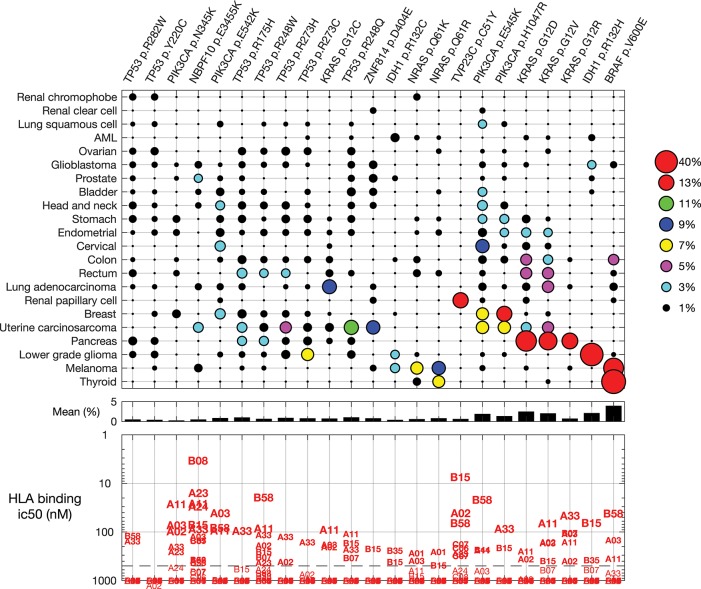
Common mutations across all indications and their predicted HLA binding. **(Top)** Non-synonymous mutations found in at least 10% of the samples in any one indication. Indications and mutations are clustered. **(Middle)** The mean frequency across indications. **(Bottom)** Predicted binding for each mutation to common HLA class I alleles.

For a warehouse (“off-the-shelf” or “prêt-à-porter”) approach for mutation-targeting neoantigen vaccines and TCRs, the mutation frequency is critical. The more frequent a mutation occurs, intra- and inter-indication, the larger the candidate patient population. Further, as immune recognition is dependent in part on presentation of the mutation by patient HLA alleles, identifying which HLA molecules bind the mutation neoantigen predicts the patient population. While, fascinatingly, there are re-occurring synonymous mutations (25), we focus here on common non-synonymous point mutations. Figure 4 shows non-synonymous mutations found in at least 10% of the samples in any one indication.

HLA allele B08, for example, is predicted to strongly bind (<10 nM) the peptide containing mutation NBPF10 p.E3455K, a mutation found in uterine carcinosarcoma and prostate tumors. Allele B15 is predicted to strongly bind mutation TVP23C p.C51Y, a frequent mutation in renal papillary cell tumors. Most of the re-occurring mutations are predicted to bind one or more common HLA allele with binding affinity 500 nM or stronger, suggesting candidate patient subsets for investigation of each re-occurring mutation.

**Figure 5:**
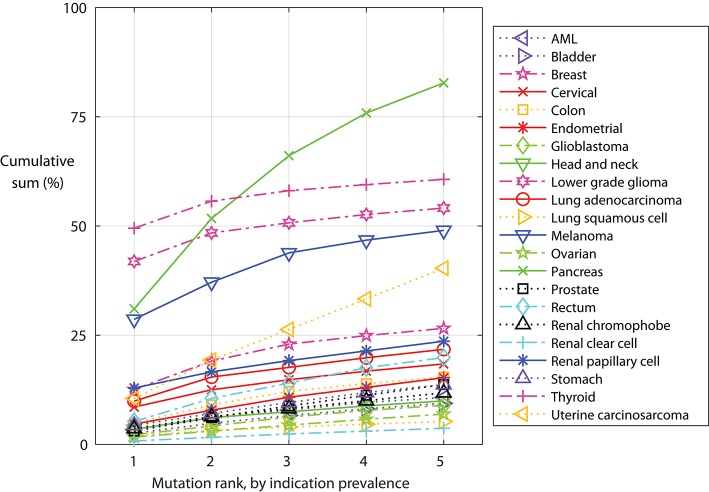
Indication-specific cumulative sum frequency of the most frequent mutations. For each indication, the mutations are ordered by frequency and the cumulative sum calculated, assuming mutations do not co-occur.

Some mutations are found in a specific indication. The IDH1 p.R132H mutation is found primarily in lower grade glioma, and found in 42% of these tumors. Other mutations, such as PIK3CA p.E545K, KRAS p.G12D, and KRAS p.G12V, occur in many indications. We examined the cumulative sum of the five most frequent mutations within an indication (Figure 5), as important for warehouse approaches. With exceptions, hot-spot mutations typically do not co-occur in one tumor clone; thus, here we assumed that mutations occur independently. When ranked from most to less frequent, the most common mutations in an indication occur in 50% (thyroid) to <1% (renal clear cell) of the tumors. Of the indications considered here, only thyroid, melanoma, pancreatic, and lower grade glioma tumors have a mutation found in more than 20% of the tumors. When examining the cumulative sum of the first five mutations, one finds that contributions of mutations two through five are large for the profiled pancreatic and uterine cancers: over 80 and 40% of the profiled pancreatic and uterine tumors, respectively, have one of the five most frequent mutations. The three most frequent mutations in pancreatic tumors are KRAS p.G12D, G12V, and G12R, demonstrating the importance of this aberration for pancreatic tumor oncogenicity. Conversely, the most frequent uterine tumor mutations are found in different genes, including TP53, PIK3CA, ZNF814, and KRAS, suggesting engagement of alternative pathways. While not reviewed here, there are re-occurring mutations in other indications, such as uveal melanoma and diffuse intrinsic pontine glioma (DIPG) tumors.

## Discussion and Conclusion

Cancer mutations are found in tumor cells and absent in non-tumorous cells. Thus, as targets, mutations are, by definition, uniquely found in the tumor cells. Some of the mutations are expressed, processed, and presented on tumor HLA molecules to T cells; those mutation-containing peptides that are recognized by T cells are neoantigens. A goal of immuno-oncology is to induce recognition of these tumor-specific, non-self targets. The number of mutations, and particularly the number of clonal immunogenic mutations, predicts tumor response to immune-strengthening therapeutics, such as anti-CLTA4 and anti-PD(L)1 mAbs (26, 27). Tumors with exceptionally high mutational burden respond favorably to immune-strengthening: pembrolizumab, an anti-PD-1 mAb, has been approved to treat MSI-H or mismatch repair deficient solid tumors, regardless of tumor site or histology (28). Thus, Figure 1, and the subclasses in Figures 2, 3, identifies the tumors and indications—those with higher mutation burden—potentially more likely to respond to general immune strengthening agents (those agents not targeting specific mutations).

Patient-specific mutations can be targeted using rapidly manufactured, individualized therapeutic vaccines. Mutations are identified in a patient's tumor using next-generation sequencing and bioinformatics, prioritized for vaccine inclusion using criteria including mutation clonality (27) and peptide HLA binding affinity (14), manufactured, and administered with an adjuvant, potentially as part of combination therapy. Several companies have individualized clinical trials underway, including Advaxis (NCT03265080), Agenus (NCT03673020), BioNTech/Genentech (NCT03289962), Gritstone (NCT03639714), and Neon (NCT03597282).

In contrast to patient-specific individualized neoantigens, shared neoantigens provide the opportunity to create warehouse vaccines and TCRs for prêt-à-porter application. Protein post-translational modifications have been identified that are tumor-specific, shared across multiple tumors, and immunogenic (29), and thus classify as warehouse targets. Here, we examine the frequency and immunogenicity of protein-modifying DNA point-mutations found in the TCGA cohorts.

As expected, there is not a single neoantigen—no magic bullet—that is found in all tumors in all indications and for all HLA alleles. However, there are mutations found frequently in specific indications, such as IDH1 p.R132H in lower grade glioma. BRAF p.V600E, KRAS p.G12D, and KRAS p.G12V are frequent in multiple indications. Other mutations, such as PIK3CA p.E545K, are less frequently found in a single indication but are found in the tumors of many indications.

Re-occurring mutations usually do not co-occur in a single tumor clone. The five most frequent mutations in each indication typically account for more than 30% of the tumors in pancreatic, thyroid, lower grade glioma, melanoma, and uterine cancers. Presentation of each mutation-containing peptide is HLA dependent and, as such, a warehouse TCR or vaccine targeting a re-occurring mutation will be relevant for only a subset of patients. Thus, the warehouse must be stocked with multiple vaccines or TCRs, accounting for both the tumor variation and patient HLAs. Indeed, some mutations will be more visible to some patients, depending on their HLA alleles. Indeed, recent work suggests that an individual's HLA alleles shape the allowable common mutations that can occur in the individual (30), further confirming that common mutations can be seen by the immune system.

Using the impressive public domain TCGA dataset, this work shows the presence of non-synonymous single-nucleotide mutations across a broad panel of tumor indications and potential immunotherapy application. As previously described, melanoma and lung cancers have higher numbers of mutations relative to other tumors. These indications also have a long tail: a population of tumors with an exceptionally high number of mutations. Organs at risk for MSI tumors, including colon, stomach, and endometrial, show similar mutation distributions, comprising a core group of tumors with fewer mutations, typically the micro-satellite stable tumors, and the long tail of high mutation MSI tumors. Mutation rates vary among molecularly-defined tumor sub-groups: breast basal tumors have, on average, more mutations than luminal A tumors. Smoking and TP53 mutations are associated with high tumor mutation burden in lung cancers. Finally, re-occurring mutations can be found in the profiled tumors: BRAF p.V600E is found in many thyroid tumors and melanomas and mutations such as PIK3CA p.E454K can be found at appreciable levels across multiple indications. HLA binding of non-self peptides is essential for neoantigen generation; that many of these mutation-containing peptides are predicted to bind common HLA alleles increases the likelihood that they are bona fide neoantigens suitable for warehouse vaccines and T-cell therapies.

## Author Contributions

JC initiated the study and generated the figures. MU, SP, and JC collected and analyzed the data. JC, RS, and JB wrote the manuscript.

### Conflict of Interest Statement

All authors are, or have previously been, employed by Agenus Inc. to develop cancer immunotherapies.
